# VI Scandinavian COPD Research Symposium Published with support from Boehringer Ingelheim

**DOI:** 10.3402/ecrj.v2.30020

**Published:** 2015-11-05

## Foreword 2014 01

### Abstracts and proceedings from VI Scandinavian COPD Research Symposium

#### Magnus Sköld ^1^, Johny Kongerud^2^, Thomas Ringbaek^3^ and Terttu Harju^4^

magnus.skold@ki.se

^1^Department of Medicine, Karolinska Institutet, Stockholm, Sweden; ^2^Department of Respiratory Medicine, Rikshospitalet, Oslo, Norway; ^3^Department of Medicine, Hvidovre Hospital, Copenhagen, Denmark; ^4^Department of Pulmonary Medicine, University of Oulu, Oulu, Finland

#### 

On 21–22 November 2014, the sixth Scandinavian COPD Research Symposium was held at Holmenkollen Park Hotel Rica, Oslo. Like the previous five meetings (1–5), the purpose was to let young scientists from Denmark, Finland, Norway, and Sweden come together and present their current COPD-related research. The meeting should also facilitate collaboration and stimulate further research in the field of COPD. Eleven young scientists from our countries presented their latest data and three state-of-the-art lecturers covered two sessions: ‘Inflammation and structural changes in COPD’ and ‘COPD not caused by cigarette smoke’. To better facilitate interactions in a smaller format, three ‘break-out sessions’ was also arranged. The meeting was generously supported by grants from Boehringer Ingelheim, which also made publication of this supplement to *European Clinical Respiratory Journal* possible.

**References**

1. Sköld M, Bakke P, Kongerud J, Ringbaek T. Scandinavian COPD research symposium. Respir Med. 2005; 99: 918–25.

2. Sköld M, Bakke P, Ringbaek T. Abstracts and proceedings from II Scandinavian COPD research symposium. Respir Med COPD Update. 2007; 3: 119–27.

3. Sköld M, Kongerud J, Ringbaek T, Pietinahlo A. Proceedings and Abstracts from III Scandinavian COPD Research Symposium. Respir Med. 2009; 103(Suppl 1): S1–7.

4. Sköld M, Kongerud J, Ringbaek T, Myllärniemi M. Abstracts and Proceedings from IV Scandinavian COPD Research Symposium. Clin Respir J. 2011; 5(Suppl 1): 1–10.

5. Sköld M, Kongerud J, Ringbaek T, Myllarniemi M. Abstracts and Proceedings from the V Scandinavian COPD Research Symposium. Respir Med. 2013; 107: S1–7.

## ABSTRACTS STATE OF THE ART 02

### Inflammation in COPD

#### Dirkje S. Postma

d.s.postma@med.umcg.nl
Department of Pulmonary Medicine and Tuberculosis, University Medical Center Groningen, Groningen, The Netherlands

#### 

COPD reflects airway obstruction due to an inflammatory and remodelling process in the airways and lungs that becomes a symptomatic disease at adult age. It is caused by cigarette smoking or other environmental factors, and recently even pesticides were reported of importance. Inflammatory cells play a role in COPD development and progression and it generally accepted that neutrophils and CD8+ lymphocytes are higher in sputum and airway wall biopsies of the large airways in smokers with than without COPD. Moreover, increased numbers of macrophages populate the airways. Neutrophils are particularly found in the mucous glands and their factors likely contribute to increased mucus hypersecretion. During exacerbations increased numbers of eosinophils have been reported. There exists even a subset of COPD patients with blood eosinophilia and this has been suggested to predict a good response to inhaled steroid treatment to prevent exacerbations. In the peripheral airways and lung tissue, neutrophil, lymphocyte, and mast cell numbers are increased, next to a B cell response in the large and small airways; plasmacytoid dendritic cell numbers in the pulmonary lymphoid follicles are increased. Recently, the epithelial cells have become a target of interest given their pro-inflammatory and inflammatory function and possible role in COPD as a barrier against inhaled toxic substances. Besides local effects on the airway wall, inflammation also increases stretching and ultimately the loss of alveolar septal attachments to the small airways with emphysema as a consequence. Inflammation can also be defined by the production of cytokines, proteases, and so on. The cytokines currently thought of importance include (TNF)-α, INF-γ, interleukin (IL)-1β, IL-6, IL-17, IL-18, IL-32, TSLP, and growth factors such as TGF-β and VEGF. Given the complex nature and intricate pathways leading to the different forms of COPD gene expression profiling of lung tissues and specific cell types and other techniques to define pathways of inflammation and repair in COPD are of utmost importance. Only by integratively combining approaches of clinical sub-phenotyping, genetic, epigenetic, and proteomic approaches, we will make a next step in understanding the inflammatory mechanisms of tissue breakdown and repair associated with COPD.

## 03

### Environmental exposures and COPD

#### Paul K. Henneberger

pkh0@cdc.gov
National Institute for Occupational Safety and Health Centers for Disease Control and Prevention, Morgantown, WV, USA

#### 

Chronic obstructive pulmonary disease (COPD) is responsible for substantial morbidity and mortality worldwide. Cigarette smoking is the leading cause, but its dominance in industrialised countries has waned as smoking rates have declined. Occupational exposures continue to be important contributors to the burden of COPD. Moreover, the identification of harmful occupational exposures offers the possibility that reduction or elimination of such exposures could prevent disease. This presentation will review the major occupational causes of COPD as revealed primarily by epidemiologic studies. The summary will emphasise findings from Scandinavian research, including population-based studies and investigations conducted in specific occupations and industries. Methods for the characterisation of occupational exposures in these studies include self-reports and job exposure matrices, as well as workplace measurements of airborne dusts and gases. Different approaches have also been used to determine COPD status, such as spirometry, self-reported symptoms and disease, and cause-specific mortality. Scandinavian researchers have published multiple papers about COPD among construction, cement, smelter, and tunnel workers, but they have also identified many other occupations and industries with an elevated prevalence or incidence of obstructive disease. A topic addressed in several studies is the effect of occupational exposure by smoking status, with some results suggesting that non-smokers have a greater relative risk for occupational COPD than their co-workers who smoke. Interventions to reduce exposures and prevent COPD will be discussed.

## 04

### Early life origin of COPD

#### Andrew Bush

A.Bush@rbht.nhs.uk
Imperial College, National Heart and Lung Institute, Royal Brompton & Harefield NHS Foundation Trust, London, UK

#### 

The key questions are 1) what is COPD; 2) what is early life?; and 3) are they connected? *What is COPD?* Modern definitions rely heavily on an FEV1/FVC ratio of <70%, ignoring the developmental absurdities of a fixed ratio and the fact that many diseases cause airflow obstruction [Thorax 2012;67:88–9]. This presentation will refer to the early life origin of premature airflow obstruction. *What is early life?* Any meaningful definition must think before early childhood, to antenatal events, and even transgenerational influences on childhood development. *What is the connection?* Normal lung growth requires the child to be born with normal airway function; for lung growth in childhood to go at a normal pace; and for there to be no premature lung aging. Many COPD patients in fact have a normal rate of lung aging, underscoring the importance of early life events causing premature airflow obstruction [*NEJM* 2011; 365: 1184–92; *Am J Respir Crit Care Med* 2011; 184: 1015–51]. Transgenerational, antenatal, early postnatal and late childhood and adult factors impacting normal lung growth will be reviewed. In particular, five childhood factors have been shown to influence COPD risk at least as much as heavy smoking in adulthood [*Thorax* 2010; 65: 14–20], namely maternal, paternal and childhood asthma, maternal smoking and childhood respiratory infections. The Melbourne cohort have recently reported that nearly 50% of children with severe asthma have COPD, independent of smoking, and with no accelerated decline in lung function [Tai A, Thorax, epub]. Chronic lung disease of prematurity will also be a cause of premature airway obstruction, but the airway pathology is very different from asthma; so is the ‘COPD’ resulting from these two diseases the same, and is it the same as that in term, non-atopic smokers? *Conclusions*: If premature airflow obstruction is to be prevented, efforts must be focussed antenatally and in the pre-school years. We must not assume that all chronic airflow obstruction is the same disease.

## ABSTRACTS OF OTHER LECTURES 05

### Potential biomarkers of asthma-COPD overlap syndrome

#### Jing Gao^1^, Tarja Laitinen^2^, Hiroshi Iwamoto^3^, Harri Alenius^4^, Witold Mazur^1^ and Ville Pulkkinen^1^

jing.gao@helsinki.fi

^1^Department of Medicine, Pulmonary Division, University of Helsinki and Helsinki University Central Hospital, Helsinki, Finland; ^2^Department of Pulmonary Diseases, Turku University Central Hospital, Turku, Finland; ^3^Department of Molecular and Internal Medicine, Graduate School of Biomedical Sciences, Hiroshima University, Hiroshima, Japan; ^4^Unit of Systems Toxicology, Finnish Institute of Occupational Health, Helsinki, Finland

#### 

*Background*: The underlying inflammatory mechanisms of asthma-COPD overlap syndrome (ACOS) are poorly characterised. There is a clear unmet need to find novel biomarkers for clinical assessment and management of ACOS. We have investigated two panels of potential sputum and/or plasma biomarkers for ACOS phenotype: COPD-related biomarkers and asthma-related biomarkers. The four COPD-related biomarker are surfactant protein A (SP-A), soluble receptor for advanced glycation end-products (sRAGE), myeloperoxidase (MPO), and neutrophil gelatinase-associated lipocalin (NGAL). The four asthma-related biomarkers are periostin and interleukin 13 (IL-13), chitinase-like protein (YKL-40), and interleukin 6 (IL-6). *Aim*: To clarify the similarity and differences between asthma, COPD, and ACOS; and to identify reliable, specific, and sensitive biomarkers for ACOS. *Method*: Induced sputum and/or plasma levels of biomarkers were measured by ELISA/Luminex assays in 132 study subjects into five groups: non-smokers (*n*=24), smokers (*n*=23), asthma (*n*=32), COPD (*n*=39), and ACOS (*n*=14). *Results*: In patients with ACOS, plasma sRAGE levels were reduced whereas, the other measured biomarkers were elevated when compared with non-smokers. However, sputum NGAL, YKL-40, and IL-6 were significantly elevated in ACOS and could differentiate ACOS from asthma and COPD, and further independently with reduced lung function in the multivariate stepwise analysis. *Conclusion*: Elevated sputum levels of NGAL, YKL-40, and IL-6 are characteristic for ACOS and are related to enhanced neutrophilic and/or eosinophilic airway inflammation, remodelling, and airway obstruction. Monitoring of sputum biomarkers could be useful in clinical practice for identifying ACOS.

**References**

1. Iwamoto H*, Gao J*, Koskela J, Kinnula V, Kobayashi H, Laitinen T, et al. Differences in plasma and sputum biomarkers between COPD and COPD-asthma overlap. Eur Respir J. 2014; 43: 421–9. *Co-first authors.

2. Iwamoto H, Gao J, Pulkkinen V, Toljamo T, Nieminen P, Mazur W. Soluble receptor for advanced glycation end-products and progression of airway disease. BMC Pulm Med. 2014; 14: 68.

## 06

### Adaptive immune responses in COPD largely depend on current smoking status and not airway obstruction

#### H. Forsslund, M. Yang, M. Mikko, J. Grunewald, J. Wahlström, Å. M. Wheelock and C. M. Sköld
helena.forsslund@gmail.com
Respiratory Medicine Unit, Department of Medicine, Karolinska Institutet, Stockholm, Sweden

#### 

T cells are important cells in smoking-induced inflammation and COPD. We have previously shown that the distribution of T-cell subtypes in BAL and blood is more related to current smoking status than airway obstruction, at least in GOLD stage I–II (1). More recently, gender differences have been gaining increased attention (2). Many factors, such as symptoms and mortality rate, differ between men and women and for unknown reasons, women are more susceptible to develop the disease. We studied the distribution on T-cell subsets along with chemokines and its receptors, which are important for the recruitment of T cells to the lung. Thirty-seven never-smokers, 38 smokers with normal lung function, and 32 patients with COPD, GOLD stage I–II (23 smokers and 9 ex-smokers) underwent bronchoalveolar lavage (BAL). Using multicolour flow cytometry, BAL and blood T cells were analysed for the expression of CD4 and CD8 and the chemokine receptors CXCR3, CCR4, and CCR5. The levels of chemokines, as well as a number of cytokines and growth factors, were analysed using Luminex bead-based technology. Smokers with or without COPD had higher percentages of CD8 T cells in BAL than did never-smokers and ex-smokers with COPD (*p*<0.01 for both). Conversely, the percentages of CD4 T cells were lower in both smoking groups than in the non-smokers (*p*<0.001 for both). Furthermore, VEGF, IL-12, and IL-13 were lower in both groups of smokers compared to never-smokers and COPD ex-smokers. In addition, female smokers with normal lung function had higher levels of inflammatory mediators, including the chemokine CXCL10 and the cytokine IL-13 compared to males, indicating a gender difference in the inflammatory response to cigarette smoke. Smoking has a larger impact on the adaptive immune response than airway obstruction *per se* and there seems to be gender difference mediators important for cell recruitment. Ongoing multivariate analysis will help us combine our findings with clinical data.

**References**

1. Forsslund H, Mikko M, Karimi R, Grunewald J, Wheelock ÅM, Wahlström J, et al. Distribution of T cell subsets in bronchoalveolar lavage fluid of patients with mild to moderate COPD is dependent on current smoking status and not airway obstruction. Chest. 2014; 145: 711–22.

2. Kohler M, Sandberg AS, Kjellqvist S, Thomas A, Karimi R, Nyrén S, et al. Gender differences in the bronchoalveolar lavage cell proteome of patients with COPD. J Allergy Clin Immunol. 2013; 131: 743–51.

## 07

### COPD and vitamin D status

#### M. Moberg, T. Ringbaek, J. Vestbo, L. Ferrucci, S. Ladelund, P. Lange, G. Martinez, N. B. Roberts, E. P. A. Rutten, M. A. Spruit, J. E. A. Williams and E. F. Wouters
miamoberg@hotmail.com
University of Copenhagen, Hvidovre University Hospital, Copenhagen, Denmark

#### 

Vitamin D deficiency is common in patients with COPD. Vitamin D deficiency has been associated with increased risk of inflammation, infection, and mortality. However, it is unknown whether vitamin D deficiency is specifically associated with COPD disease mechanism or whether it is a general marker of poor health. We wanted to investigate the prognostic value of vitamin D in patients with stable COPD and in patients hospitalised due to acute exacerbation of COPD, to examine the relation between vitamin D and COPD phenotypes and to examine the relation between vitamin D deficiency and airflow limitation in a population of older subjects. Studies I and II: a cohort of patients from the pulmonary rehabilitation program at Hvidovre Hospital. Study I: 674 patients were included. Study II: 423 patients were included. Study III: a subgroup of 91 patients from the Evaluation of COPD Longitudinally to Identify Predictive Surrogate Endpoints study. Study IV: 89 patients were hospitalised due to acute exacerbation of COPD at Hvidovre Hospital. Study V: 897 subjects from the Baltimore Longitudinal Study of Aging. Vitamin D predicted neither hospitalisation nor mortality. We did not find any association between COPD phenotypes and vitamin D status. Airflow limitation was not an independent determinant of vitamin D deficiency. Our results do not support a role for vitamin D as a predictor in COPD as other clinical characteristics seem to be more important. Vitamin D deficiency is possibly representing poor health rather than being specific to COPD disease mechanism.

## 08

### Selective inhibition by simvastatin of IRF3 phosphorylation and TSLP production in dsRNA-challenged bronchial epithelial cells from COPD donors

#### A. Brandelius ^1^, I. Mahmutovic Persson^1^, J. Calvén^1^, L. Bjermer ^2^, C. G. Persson^2^, M. Andersson^2^ and L. Uller^1^

angelica.brandelius@med.lu.se

^1^Department of Experimental Medical Science, Lund University, Sweden; ^2^Department of Respiratory medicine, Lund University Hospital, Sweden

#### 

Possibly reflecting anti-inflammatory properties, statin treatment may ameliorate COPD exacerbations (1, 2). Viral infections are common triggers of exacerbations in COPD and cause Th2-type inflammation (3). We have shown that thymic stromal lymphopoietin (TSLP), a cytokine linking innate and adaptive immunity and switching on Th2-type inflammation, is overproduced in virally- and dsRNA-stimulated cells from patients with GOLD stage IV COPD (4). In this study, we test the hypothesis that simvastatin inhibits dsRNA-induced TSLP in human bronchial epithelial cells. Primary human bronchial epithelial cells (HBECs), obtained by fibre optic bronchoscopy from COPD (GOLD stage II) (*n*=7) and healthy smokers (*n*=8) individual donors, were grown until confluent in 12-well plates. The cells were stimulated with the synthetic viral surrogate molecule dsRNA (10 µg/ml) for 3 and 24 hours to induce cytokine gene expression (analysed by RT-qPCR) and protein release and production (analysed by ELISA). Simvastatin (0.2–5 µg), alternatively dexamethasone, was added prior to dsRNA. dsRNA induced TSLP, TNFα, CXCL8, and IFNβ. TSLP was overproduced in dsRNA-exposed COPD cells compared with control. Simvastatin, but not dexamethasone, inhibited dsRNA-induced TSLP. Unexpectedly, simvastatin did not affect dsRNA-induced NFκ-B activation nor did it reduce the production of TNFα and CXCL8. Instead, simvastatin inhibited dsRNA-induced IRF3 phosphorylation and generation of IFNβ. The pharmacology of simvastatin may unravel paths of inhibition of TSLP production in COPD epithelium.

**References**

1. Blamoun AI, Batty GN, DeBari VA, Rashid AO, Sheikh M, Khan MA. Statins may reduce episodes of exacerbation and the requirement for intubation in patients with COPD: evidence from a retrospective cohort study. Int J Clin Pract. 2008; 62: 1373–8.

2. Bartziokas K, Papaioannou AI, Minas M, Kostikas K, Banya W, Daniil ZD, et al. Statins and outcome after hospitalization for COPD exacerbation: a prospective study. Pulm Pharmacol Ther. 2011; 24:625–31.

3. Papi A, Luppi F, Franco F, Fabbri LM. Pathophysiology of exacerbations of chronic obstructive pulmonary disease. Proc Am Thorac Soc. 2006; 3: 245–51.

4. Calvén J, Yudina Y, Hallgren O, Westergren-Thorsson G, Davies DE, Brandelius A, et al. Viral stimuli trigger exaggerated thymic stromal lymphopoietin expression by chronic obstructive pulmonary disease epithelium: role of endosomal TLR3 and cytosolic RIG-I-like helicases. J Innate Immun. 2012; 4: 86–99.

## 09

### The role of inflammasomes in pulmonary hypertension associated with COPD

#### F. T. Cero ^1,2,3^, V. Hillestad^2,3^, I. Sjaastad^2,3^, A. Yndestad^3,4,5^, P. Aukrust^4,5^, E. M. Løberg^6^, G. Christensen^2,3^, K. O. Larsen^1^ and O. H. Skjønsberg^1^

cero.fadila@medisin.uio.no

^1^Department of Pulmonary Medicine, Oslo University Hospital, Ullevål, Oslo, Norway; ^2^Institute for Experimental Medical Research, Oslo University Hospital, Ullevål, Oslo, Norway; ^3^Center for Heart Failure Research, University of Oslo, Oslo, Norway; ^4^Research Institute of Internal Medicine, Oslo University Hospital Rikshospitalet, Oslo, Norway; ^5^K.G. Jebsen Inflammation Research Centre, University of Oslo, Oslo, Norway; ^6^Department of Pathology, Oslo University Hospital, Ullevål, Oslo, Norway

#### 

*Background*: Inflammasomes are molecular platforms which are part of the innate immunity and the best characterised is the NLRP3 inflammasome consisting of the receptor (NLRP3), the adaptor protein (ASC) and caspase-1, the protease which activate IL-18 and IL-1β. Increased levels of IL-18 and IL-1β are found in the lungs of COPD patients. Uncorrected chronic hypoxemia in COPD patients is associated with the development of pulmonary hypertension. *Objective*: To study role of NLRP3 inflammasome in chronic hypoxia-induced pulmonary hypertension. *Methods*: Active caspase-1, IL-18, and IL-1β were measured by western blot analysis of lung homogenates in WT, NLRP3^−/−^ and ASC^−/−^ mice exposed to 1 months of hypoxia in a tightly sealed chamber containing 10% oxygen. Right ventricular systolic pressure (RVSP) was measured with a microtipped transducer catheter after 3 months of hypoxia exposure. *Results*: Chronic hypoxia induced increased protein levels of caspase-1, IL-18 and IL-1β in WT, and NLRP3^−/ −^ mice. However, hypoxia-induced activation of the inflammasome was absent in ASC^−/ −^ mice shown by no increase of active caspase-1, IL-18, or IL-1β. RVSP of ASC^−/ −^ exposed to hypoxia was significantly lower than WT hypoxia (40.8±1.5 mmHg versus 55.8±2.4 mmHg, *p*<0.001), demonstrating a lesser degree of pulmonary hypertension. RVSP of NLRP3^−/ −^ exposed to hypoxia was not significantly altered compared to WT hypoxia (52.4±2.3 mmHg versus 55.8±2.4 mmHg). *Conclusions*: Altogether, our results show that ASC^−/ −^ mice are protected against hypoxia-induced pulmonary hypertension, suggesting inflammasome activation to be involved in the pathogenesis of the disease.

## 10

### Emphysema progression is visually detectable in continuous but not in former smokers in a five-year time frame

#### Inter-observer analysis and time-trend of visual assessments of CT scans from The Danish Lung Cancer Screening Trial

#### Laura H. Thomsen ^1^, Mathilde M. W. Wille^1^, Asger Dirksen^1^, Jens Petersen^2^, Jesper Holst Pedersen^3^ and Saher B. Shaker^1^

laurahohwu@gmail.com

^1^Department of Respiratory Medicine, Gentofte Hospital, Copenhagen University, København, Denmark; ^2^Department of Computer Science, Copenhagen University, København, Denmark; ^3^Department of Thoracic Surgery, Rigshospitalet, Copenhagen University Hospital, København, Denmark

#### 

To evaluate interobserver agreement and time-trend in chest CT assessment of emphysema, airways, and interstitial abnormalities in a lung cancer screening cohort. Visual assessments of baseline and fifth-year scan of 1990 participants were performed independently by two observers. Results were standardised by means of an electronic score sheet; kappa and time-trend analyses were performed. We found that interobserver agreement was substantial in early emphysema diagnosis; highly significant (*p*<0.001) time-trends in both emphysema presence and grading were found (higher prevalence and grade of emphysema in late scans). Significant progression in emphysema was seen in continuous smokers, but not in former smokers. Agreement on centrilobular emphysema subtype was substantial, agreement on paraseptal subtype moderate. Agreements on panlobular and mixed subtypes were only fair. Agreement was fair regarding airway analysis. Interstitial abnormalities were infrequent in the cohort and agreement on these fair to moderate. Highly significant time-trend was found regarding interstitial abnormalities, which were more frequent in late scans. In conclusion, visual scoring of chest CT scan characterise presence, pattern, and progression of early emphysema. Continuous smokers progress, whereas former smokers do not. Early airways disease probably requires supplementary automated computer analysis. Further studies are needed on reliability in visual assessment of interstitial lung disease.

**References**

1. Barr RG, Berkowitz EA, Bigazzi F, Bode F, Bon J, Bowler RP, et al. A combined pulmonary-radiology workshop for visual evaluation of COPD: study design, chest CT findings and concordance with quantitative evaluation. COPD. 2012; 2: 151–9.

2. Gietema HA, Müller NL, Fauerbach PV, Sharma S, Edwards LD, Camp PG, et al. Quantifying the extent of emphysema: factors associated with radiologists’ estimations and quantitative indices of emphysema severity using the ECLIPSE cohort. Acad Radiol. 2011; 18: 661–71.

3. Sverzellati N, Devaraj A, Desai SR, Quigley M, Wells AU, Hansell DM. Method for minimizing observer variation for the quantification of high-resolution computed tomographic signs of lung disease. J Comput Assist Tomogr. 2011; 5: 596–601.

4. Mets OM, Smit EJ, Mohamed Hoesein FA, Gietema HA, Bokkers RP, Attrach M, et al. Visual versus automated evaluation of chest computed tomography for the presence of chronic obstructive pulmonary disease. PLoS One. 2012; 7: e42227.

5. Hansell DM, Bankier AA, MacMahon H, McLoud TC, Müller NL, Remy J. Fleischner Society: glossary of terms for thoracic imaging. Radiology. 2008; 3: 697–722.

## 11

### Combined effect of smoking and occupational exposure to dusts, gases or fumes on the incidence of COPD

#### Annette Kainu ^1^, Paula Pallasaho^2^, Anssi Sovijärvi^3,4^, Ari Lindqvist^5^ and Päivi Piirilä^3,4^

annette.kainu@helsinki.fi

^1^HUCH Heart and Lung Center, Peijas Hospital, Helsinki, Finland; ^2^Team for Control of Hypersensitivity Diseases, Finnish Institute of Occupational Health, Helsinki, Finland; ^3^Department of Clinical Physiology and Nuclear Medicine, HUS Medical Imaging Center, Helsinki University Central Hospital, Helsinki, Finland; ^4^University of Helsinki, Helsinki, Finland; ^5^Research Unit of Pulmonary Diseases, Helsinki University Central Hospital, Helsinki, Finland

#### 

To assess risk factors related to the development of chronic obstructive pulmonary disease (COPD) including smoking and occupational exposure (OE) to dusts, gases, or fumes, we performed a longitudinal 11-year follow-up postal survey. The original study population was a random population sample of 8,000 inhabitants of Helsinki aged 20–69 years in 1996. Participants of the first postal questionnaire were invited to this follow-up survey in 2007 with 4,302 (78%) answers obtained. Cumulative incidence of COPD in 11 years was 3.43% corresponding to an incidence rate of 3.17/1,000/year after exclusion of those with self-reported physician-diagnosed COPD and ever COPD in 1996. Smoking and age, but not gender, were associated with incident COPD. Reported family history of COPD increased the cumulative incidence to 8.55% versus 3.04% among those without a family history (*p*<0.001). In multivariate analysis, significant independent risk factors for incident COPD were: current smoking in 1996 (OR 4.40 [95% CI 2.89–6.71]), age over 50 (OR 3.42 [95% CI 2.22–5.26]), family history of COPD (OR 2.08 [95% CI 1.27–3.43]), ever asthma (OR 2.28 [1.35–3.86]), and self-reported OE (OR 2.14 [1.50–3.05]). Occupational exposure to dusts, gases, or fumes, assessed both based on self-reported exposure and a job exposure matrix using reported professions, was an independent risk factor for incident COPD. Smoking and OE together yielded an additive effect on incidence of COPD.

**Reference**

1. Pallasaho P, Kainu A, Sovijärvi A, Lindqvist A, Piirilä PL. Combined effect of smoking and occupational exposure to dusts, gases or fumes on the incidence of COPD. COPD. 2014; 11: 88–95. doi: http://dx.doi.org/10.3109/15412555

## 12

### Occupational exposure and CT in patients with and without COPD

#### Øistein Svanes ^1,2,3^, Trude Duelien Skorge^1^, Ane Johannessen^4^, Thomas Blix Grydeland^2^, Amund Gulsvik^3^ and Per Bakke^3^

oistein.svanes@k2.uib.no

^1^Department of Occupational Medicine, Haukeland University Hospital, Bergen, Norway; ^2^Department of Thoracic Medicine, Haukeland University Hospital, Bergen, Norway; ^3^Department of Clinical Science, University of Bergen, Bergen, Norway; ^4^Centre for Clinical Research, Haukeland University Hospital, Bergen, Norway

#### 

*Background*: There is limited knowledge on the effect of occupational exposure on quantitative CT (qCT) measurements of emphysema. *Aims*: To examine the effect of occupational exposure assessed by a job exposure matrix (JEM) on CT measurements of emphysema. *Methods*: In the Norwegian GenKOLS study 2003–2005, 951 ever-smokers (49% with COPD) aged 40–85 years performed spirometry and CT examination; of these 941 completed a full occupational history. In this study, a JEM was used to assess occupational exposure in longest held job, and CT measured emphysema (% low-attenuation areas, %LAA) was the main outcome. Multiple linear regression was used for the multivariate analyses, adjusting for sex, age, and smoking (pack years and previous/current smoker). *Results*: Multiple linear regression showed significant associations between occupational exposure to biological and dust gas/fumes in longest held job, and %LAA. In COPD subjects, the unadjusted regression coefficients for %LAA in subjects with exposure to biological dust as compared to those with no exposure were 0.67 (95% CI: 0.43–0.91). The corresponding figures for gas/fumes exposure were 0.30 (0.037–0.57). After adjustments, the associations still remained statistically significant for COPD subjects exposed to biological dust and gas/fumes; 0.29 (0.031–0.55) and 0.29 (0.037–0.54). In subjects without COPD, no relationship between occupational exposure and level of emphysema was found. *Conclusions*: COPD subjects with occupational exposure to biological dust and gas/fumes in their longest held job were found to have higher levels of CT measured emphysema compared to COPD subjects with no exposure.

## 13

### Preterm birth and lung function development

#### Per Thunqvist

per.thunqvist@sodersjukhuset.se
Department of Clinical Science and Education, Karolinska Institutet, Stockholm, Sweden; Department of Pediatrics, Sachs’ Children's Hospital, Stockholm, Sweden

#### 

Due to a tremendous development of neonatal care, the survival rate of infants born extremely preterm (<28 weeks of gestation) has more than doubled in the last decades. The majority of these infants are diagnosed with and classified for severity of bronchopulmonary dysplasia (BPD) (1). In a longitudinal cohort study, we found no association between BPD classification and level of reduced lung function using infant lung function tests at 6 and 18 months of age. However, we report on a clear association between decreased lung function and respiratory symptoms (2). At school age, we found lower lung function for all levels of BPD severity for children with BPD compared to preterm children without BDP; however, only a minority of all children reported respiratory symptoms (3). Hence, early classification of BPD severity provides limited information on forthcoming lung function. We also compared lung function in individuals born moderate-to-late preterm (gestation week 32–36) to those born at term at eight and 16 years of age. The moderate-to-late preterm group did not report increased respiratory symptoms but presented with lower lung function at both ages without catch-up growth between the two ages (4). A recently published longitudinal follow-up studies from childhood to adult life has demonstrated that reduced airway function measured during childhood is still present in early adulthood (5). Besides not reaching the same level of lung function peak, it is still unclear if former preterm individuals also are at risk of more rapid decline. Future research should aim to study the mechanism for altered lung function after preterm birth.

**References**

1. Fellman V, Hellstrom-Westas L, Norman M, Westgren M, Kallen K, Lagercrantz H, et al. One-year survival of extremely preterm infants after active perinatal care in Sweden. JAMA. 2009; 301: 2225–33.

2. Thunqvist P, Gustafsson P, Norman M, Wickman M, Hallberg J. Lung function at 6 and 18 months after preterm birth in relation to severity of bronchopulmonary dysplasia. Pediatr Pulmonol. 2015; 50: 978–86.

3. Brostrom EB, Thunqvist P, Adenfelt G, Borling E, Katz-Salamon M. Obstructive lung disease in children with mild to severe bpd. Respir Med 2010; 104: 362–70.

4. Thunqvist P, Gustafsson PM, Schultz ES, Bellander T, Berggren-Broström E, Norman M, et al. Lung function at age 8 and 16 years after moderate-to-late preterm birth. Submitted.

5. Vollsaeter M, Roksund OD, Eide GE, Markestad T, Halvorsen T. Lung function after preterm birth: development from mid-childhood to adulthood. Thorax 2013; 68: 767–76.

## 14

### COPD in never-smokers

#### Yunus Çolak ^1,2^ and Peter Lange^1,2^

yunuscol@gmail.com

^1^The Copenhagen General Population Study, Herlev Hospital, Copenhagen University Hospital, Copenhagen, Denmark; ^2^Faculty of Health and Medical Sciences, University of Copenhagen, Copenhagen, Denmark

#### 

Tobacco smoking remains to be the single most important risk factor for chronic obstructive pulmonary disease (COPD) (1). However, a substantial proportion of individuals with COPD in western countries are never-smokers, indicating that other risk factors may exist (2). A recent publication based on data from 69,000 participants in the Copenhagen General Population Study (CGPS) reports that never-smokers comprise 22% of individuals with COPD (3). Although, never-smokers with COPD had less severe disease with a better prognosis compared to ever-smokers with COPD, risk of lung-related hospital admissions were substantial. Compared to never-smokers without COPD, hazard ratios (HRs) for hospital admission with COPD were 8.6 (95% confidence interval: 5.3–14) in never-smokers with COPD, 30 (22–41) in former-smokers with COPD, and 43 (32–59) in current-smokers with COPD. HRs for hospital admission with pneumonia were 1.9 (1.4–2.6), 2.8 (2.3–3.4), and 3.4 (2.9–4.2), respectively. However, compared to never-smokers without COPD, never-smokers with COPD did not differ with regard to risk of lung cancer, cardiovascular comorbidities, and all-cause mortality. Surprisingly, never-smokers with COPD had a lower exposure to passive smoking and to occupational exposure compared to ever-smokers with COPD. These observations suggest that never-smokers with COPD constitute a different phenotype than ever-smokers with COPD. In an ongoing PhD project, we plan to investigate never-smokers with COPD in more details with special focus on the presence of undiagnosed asthma in this subgroup. In addition, we will present data on the impact of tobacco smoking on clinical characteristics and prognosis of individuals with asthma in the CGPS.

**References**

1. Vestbo J, Hurd SS, Agusti AG, Jones PW, Vogelmeier C, Anzueto A, et al. Global strategy for the diagnosis, management, and prevention of chronic obstructive pulmonary disease: GOLD executive summary. Am J Respir Crit Care Med. 2013; 187: 347–65.

2. Salvi SS, Barnes PJ. Chronic obstructive pulmonary disease in non-smokers. Lancet. 2009; 374: 733–743.

3. Thomsen M, Nordestgaard BG, Vestbo J, Lange P. Characteristics and outcomes of chronic obstructive pulmonary disease in never smokers in Denmark: a prospective population study. Lancet Respir Med. 2013; 1: 543–50.

## 15

### Epidemiological aspects of COPD among never-smokers

#### Stig Hagstad

stig.hagstad@gu.se
Krefting Research Centre, Sahlgrenska Academy, University of Gothenburg, Gothenburg, Sweden

#### 

The main risk factor for developing COPD is active tobacco smoke. However, COPD also occurs among subjects who are never-smokers. Globally, use of biomass fuel for heating and cooking as well as tuberculosis has been associated with COPD among never-smokers although the risk profile in developed countries is not yet fully established. My doctoral thesis concerns the epidemiology of COPD among never-smokers and is based on data from the Obstructive Lung Disease in Northern Sweden (OLIN) studies. The first study examined prevalence, risk factors, and symptomatology of COPD in never-smokers. We found that one in five subjects with COPD had never smoked themselves. The overall prevalence of COPD among never-smokers was 6.9%, and for GOLD stage ≥II 3.5%, compared with 14.0 and 7.3% respectively among all subjects. Respiratory symptoms (i.e. sputum production, recurrent wheeze, and dyspnoea) were highly prevalent among never-smokers with GOLD stage ≥II. Risk factor analysis found COPD among never-smokers to be significantly associated with age ≥66 years (OR 5.56, 95% CI 2.56–12.21) and a previous physician diagnosis of asthma (OR 2.96, 1.44–6.08). The subsequent study examined the role of passive smoke as a risk factor for COPD in 2,118 lifelong never-smokers. Passive smoking in various settings was associated with COPD among never-smokers in a dose-dependent manner. Exposure to passive smoke both ever at home and at both current and previous workplace was, after belonging to the oldest age group, the strongest risk factor for COPD among never-smokers (OR 3.80, 1.29–11.2).

